# The heat shock response in neurons and astroglia and its role in neurodegenerative diseases

**DOI:** 10.1186/s13024-017-0208-6

**Published:** 2017-09-18

**Authors:** Rebecca San Gil, Lezanne Ooi, Justin J. Yerbury, Heath Ecroyd

**Affiliations:** Illawarra Health and Medical Research Institute and School of Biological Sciences, University of Wollongong, Northfields Ave, Wollongong, 2522 Australia

**Keywords:** Neurodegeneration, Amyotrophic lateral sclerosis, Huntington’s disease, Proteostasis, Heat shock response, Heat shock factor 1, Motor neurons, Striatal neurons, Astroglia

## Abstract

Protein inclusions are a predominant molecular pathology found in numerous neurodegenerative diseases, including amyotrophic lateral sclerosis and Huntington’s disease. Protein inclusions form in discrete areas of the brain characteristic to the type of neurodegenerative disease, and coincide with the death of neurons in that region (e.g. spinal cord motor neurons in amyotrophic lateral sclerosis). This suggests that the process of protein misfolding leading to inclusion formation is neurotoxic, and that cell-autonomous and non-cell autonomous mechanisms that maintain protein homeostasis (proteostasis) can, at times, be insufficient to prevent protein inclusion formation in the central nervous system. The heat shock response is a pro-survival pathway induced under conditions of cellular stress that acts to maintain proteostasis through the up-regulation of heat shock proteins, a superfamily of molecular chaperones, other co-chaperones and mitotic regulators. The kinetics and magnitude of the heat shock response varies in a stress- and cell-type dependent manner. It remains to be determined if and/or how the heat shock response is activated in the different cell-types that comprise the central nervous system (e.g. neurons and astroglia) in response to protein misfolding events that precede cellular dysfunctions in neurodegenerative diseases. This is particularly relevant considering emerging evidence demonstrating the non-cell autonomous nature of amyotrophic lateral sclerosis and Huntington’s disease (and other neurodegenerative diseases) and the destructive role of astroglia in disease progression. This review highlights the complexity of heat shock response activation and addresses whether neurons and glia sense and respond to protein misfolding and aggregation associated with neurodegenerative diseases, in particular Huntington’s disease and amyotrophic lateral sclerosis, by inducing a pro-survival heat shock response.

## Background

Neurodegenerative diseases (NDs), such as amyotrophic lateral sclerosis (ALS) and Huntington’s disease (HD), are thought to manifest through either a loss of function of the wild-type (WT) protein or toxic gain-of-function as a result of its oligomerization and aggregation [1]. The neuron-specific degeneration observed in discrete regions of the brain in NDs suggests that specific neuronal sub-types are particularly vulnerable to protein misfolding and aggregation. This may be the result of: (i) a post-mitotic inability to dilute toxic protein species through cell-division; (ii) an age-related decline in the systems that maintain protein homeostasis (proteostasis); (iii) a failure of the proteostasis network to detect pathogenic protein aggregates; or (iv) a combination of these factors [2–4].

Molecular chaperones are a central component of the proteostasis network as they act to facilitate the correct folding of nascent polypeptides, maintain partially-folded protein intermediates in folding-competent states and re-fold damaged proteins [5–8]. A recent comprehensive analysis of the human “chaperome” identified 332 chaperone genes, 142 of which correspond to the heat shock protein subfamilies (Hsp90, Hsp70, Hsp60, Hsp40 and sHsps) [4]. The Hsps are a family of evolutionarily conserved chaperones with diverse functions in proteostasis. In addition to their well characterized molecular chaperone functions, Hsps also stabilize the cytoskeleton, regulate stress responses, mitigate apoptotic signalling and shuttle damaged proteins for degradation by the ubiquitin proteasome system or by autophagy [9, 10].

There is a dramatic up-regulation of Hsp expression in cells upon induction of the heat shock response (HSR). The activation of this pathway is a primary defense mechanism that protects cells from stress conditions that promote protein misfolding, aggregation and cell death. It has previously been shown that components of the HSR may be neuroprotective due to their ability to interact with the earliest misfolded proteins that trigger pathogenic aggregation. Indeed, numerous studies have shown that Hsps can prevent the aggregation of various disease-associated proteins in vitro*,* for example, mutant superoxide dismutase 1 (SOD1) in ALS [11–15]. Heat shock proteins can also interact with pathogenic proteins in vivo and have been found co-localized with plaques and inclusions in transgenic mouse models of NDs and patient post-mortem tissues [16–19]. For example, Hsc70 was co-localized with inclusion bodies in spinal cord sections of SOD1^G93A^, SOD1^G85R^, and SOD1^G37R^ transgenic mice, and human sporadic ALS cases [17]. The co-localization of Hsps with inclusions suggests that Hsps are diverted into inclusions and therefore unavailable to perform normal “housekeeping” functions.

Little is known about how and/or if the HSR is induced in neuronal and glial cells by pathogenic protein aggregation. Elucidating whether the HSR is triggered by protein aggregation and, if so, the mechanism(s) by which this occurs, is important for future work aimed at developing proteostasis-modulating therapeutics to ameliorate ND pathologies. The objectives of this review are to summarize what is currently known about the activation of the HSR in different tissues and cell-types during cellular stress, and explore evidence regarding the involvement of the HSR in rescuing neurons and astroglia from pathological stress associated with NDs. Due to the diversity in how different NDs manifest in the CNS (i.e. dysfunction of different neuronal and non-neuronal cell types across different brain regions), this review seeks to provide a comprehensive summary of the literature surrounding the HSR in cells associated with HD and ALS. We conclude by drawing correlations between our core findings in the HD and ALS literature with NDs in general. In doing so, progress in this field of research is evaluated, gaps in our knowledge are highlighted and possible solutions are discussed.

## The HSR

Proteotoxic cellular insults have a common effect of damaging proteins and inducing the accumulation of partially-folded protein intermediates. This in turn can activate transcription factors and induce the HSR. The human genome encodes four heat shock transcription factors (HSF), HSF1 – HSF4, which have unique and overlapping functions [20]. Heat shock transcription factor 1 is the prime integrator of transcriptional responses during stress and is responsible for the induction of the HSR. The role of HSF1 in the activation and attenuation of the HSR in cells under conditions of cellular stress is discussed briefly below.

### Heat shock transcription factor 1

**Fig. 1:**
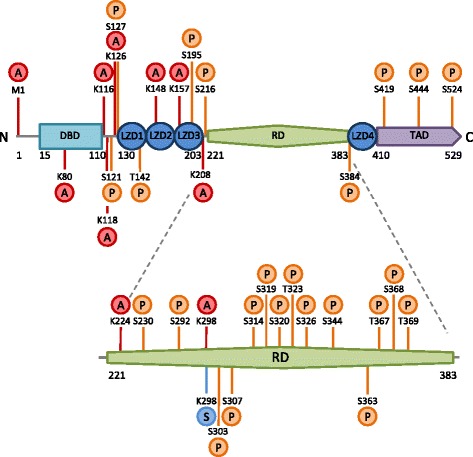
Post-translational modifications of HSF1 in relation to the functional domains in the protein. The HSF1 protein consists of a DNA-binding domain (DBD), four leucine zipper domains (LZD), a regulatory domain (RD) and a transactivating domain (TAD). The proposed sites of serine/threonine phosphorylation (P), lysine acetylation (A) and phosphorylation-dependent lysine SUMOylation (S) are marked on the HSF1 amino acid chain. These post-translational modifications are mediated by numerous kinases, acetylases and SUMOylases and act to modulate the stabilization and activity of HSF1 and thus the strength and duration of the HSR

Heat shock transcription factor 1 is constitutively expressed in most tissues and cell types and, apart from its role in the HSR, is involved in a wide range of processes including organismal development, insulin signaling and cancer metastasis (for recent comprehensive reviews see [21, 22]). Post-translational modifications are critical in modulating the activity of HSF1 [23]: it can be acetylated [24, 25], SUMOylated [26] and extensively phosphorylated [27] (Fig. 1). The type and site of each post-translational modification have been predominantly identified by proteomic mass spectrometry and site-directed mutagenesis experiments [22, 23, 28]. Whilst the activation of HSF1 is complex and only partially understood, previous studies highlight the importance post-translational modifications play in stabilising, activating and inhibiting the transcriptional activity of HSF1 [28]. For example, conversion of HSF1 into a transcriptionally active trimer occurs concurrently with extensive post-translational modifications including stress-inducible hyperphosphorylation of S230, S326 and T142 [29–31], such that hyperphosphorylated HSF1 is used as a marker of HSF1 activation [32–35]. Aside from (extensive) phosphorylation, acetylation and SUMOylation of HSF1 also play important roles in regulating the strength and duration of the HSR (for comprehensive reviews see [21, 36]).

### HSR activation and attenuation

**Fig. 2:**
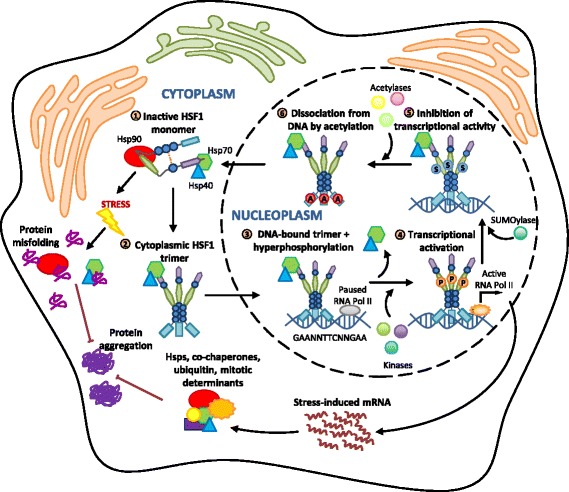
The activation of HSF1 and its binding to DNA is regulated by a multi-step pathway that involves nuclear accumulation, intramolecular and intermolecular protein interactions, and post-translational modifications. (1) In the absence of stress, HSF1 is maintained in a monomeric state through the regulatory actions of several post-translational modifications, intramolecular contacts, and interactions with Hsps in inhibitory complexes. Cellular stress results in the accumulation of misfolded and damaged proteins, which compete with HSF1 for binding to Hsps. (2) HSF1 monomers are released and undergo a conformational change conducive to trimerization. (3) Concurrent nuclear accumulation, HSE-binding and hyperphosphorylation of trimeric HSF1 occur. (4) This process releases RNA PolII from a paused to an active state to initiate the transcription of stress-induced genes. (5) SUMOylation at K298 and binding of Hsp40/Hsp70 represses the transcriptional activity of HSF1 trimers. (6) Acetylation at K80 disrupts HSF1 binding to DNA and HSF1 trimers dissociate and re-join the monomeric pool in the cytosol. Stress-inducible Hsps participate in a negative-feedback loop to inhibit further HSF1 activation

Under conditions of cellular stress, HSF1 monomers form activated homo-trimers and translocate into the nucleus. Trimerization is mediated through the formation of leucine zippers on adjacent HSF1 oligomerization domains (Fig. 2) [37, 38]. Activated HSF1 trimers bind to *cis*-regulatory elements on DNA composed of nGAAn pentamers (where n is any base), collectively called heat shock elements [39–42]. The extent and duration of HSF1-mediated transcription is influenced by the number of heat shock elements, the exact sequence of nGAAn pentamers in the promoter regions of HSF1 target genes, and post-translational modifications on HSF1 itself [21]. In addition to the rapid up-regulation of Hsp expression in response to cellular stress, HSF1 also coordinates the expression of many transcriptional and translational regulators, co-chaperones, ubiquitin, signaling molecules and mitotic regulators [21, 43, 44].

Heat shock factor 1-mediated transcription is attenuated by an auto-regulatory mechanism, whereby HSF1-induced Hsps competitively inhibit HSF1 trimer activity [22, 28]. This negative feedback loop provides an important mechanism by which cells can regulate the activation and attenuation of an HSR via the presence and concentration of Hsps in the cell. The trimerisation of HSF1 is suppressed by interaction of the monomer with a multi-chaperone complex composed of Hsp90, co-chaperone p23 and immunophilin FK506-binding protein 5 [45, 46]. In addition, the chaperonin TRiC/CCT can also interact with HSF1 to inhibit its activation [47]. Active HSF1 trimers in the nucleus can be inhibited by the binding of Hsp70 and its co-chaperone Hsp40, possibly through recruitment of the Hsp70-interacting transcriptional co-repressor, CoREST [48, 49]. Further characterization of the mechanisms that modulate HSF1 activity (and therefore, the HSR) may help elucidate additional targets for therapies that could be used to boost the HSR in the context of disease such as NDs.

## The HSR in the mammalian CNS

In mammals, the HSR varies both in terms of kinetics (i.e. how fast stress-induced transcripts are generated) and magnitude (i.e. the fold increase in Hsp levels) between tissues and even between cells in the same tissue [50–52]. Analysis of the human “chaperome” shows that the constitutive expression of housekeeping chaperones and co-chaperones varies between tissues [52]. For example, sHsps are overrepresented in skeletal and cardiac muscle compared to the brain [52]. With regard to inducing the HSR, whole organism hyperthermia results in an HSR that varies substantially across tissues types [50]. For example, in heat-stressed rats, the kinetics of the response and magnitude of induction where found to vary between the brain, liver and skin when assessed over time by northern blot of Hsp70 and Hsp27 stress-induced mRNA [50]. Moreover, cells in the same tissue can display different capacities to induce the HSR. For example, cultured astrocytes elicit faster and higher levels of Hsp70 expression after heat shock (45 °C for 30 min) compared to cultured cortical neurons [51]. These studies strongly suggest that the capacity of a cell to sense stress and elicit an HSR differs in a cell-type dependent manner.

An attenuated HSR may be an intrinsic characteristic of neurons [35, 53–55]. Indeed, this hypothesis is supported by several animal studies that have challenged rodents with hyperthermia or ischemic injury and shown that neurons do not induce Hsp70 expression after exposure to these stressors, whereas surrounding astroglial cells do [35, 51, 54–59]. These seminal studies show that under conditions of acute cellular stress, neurons are inherently poor activators of the HSR, whereas astroglia readily activate the HSR. This prompts the question of whether neuronal and astroglial cells respond to chronic ND-relevant stressors (such as protein aggregation) and, if so, whether there is a difference in the response of neuronal and astroglial cells to this stress. In an attempt to address these questions, the remainder of this review deals with what is known about HSR activation in the context of two NDs, HD and ALS.

### Huntington’s Disease

Huntington’s disease is characterized by the formation of intracellular inclusions composed primarily of the ubiquitous protein huntingtin (Htt) and by the subsequent death of striatal medium spiny neurons and cortical pyramidal neurons [60, 61]. The genetic basis for the pathogenesis of HD is a CAG codon repeat expansion in the Huntingtin (*HTT*) gene, which leads to a mutant protein that contains an expanded poly-glutamine (polyQ) sequence [62]. Expansions of the polyQ tract beyond 36 glutamines result in an aggregation-prone Htt protein; the length of the polyQ-expansion correlates with the age of disease onset and severity (i.e. the longer the polyQ tract, the earlier the onset of disease and the more rapid the disease progression) [63]. Well-characterized cell and transgenic mouse models of HD have been used in investigations into the impact of the HSR on the pathogenicity of polyQ-expansions.

PolyQ-expanded Htt may interact differently with the proteostasis network compared to other disease-associated proteins. Evidence for this comes from studies that have shown that polyQ-expanded Htt^103Q^ (i.e. Htt with a polyQ stretch of 103 glutamines) partitions exclusively into perivacuolar inclusion bodies (IPODs: Insoluble PrOtein Deposits) that store terminally aggregated proteins [64]. Fluorescence recovery after photobleaching experiments showed that Htt^103Q^ proteins sequestered into IPODs are immobile [65] indicating that sequestration into IPODs prevents possible interactions of the aggregated protein with components of the proteostasis network, exacerbating the formation of inclusions in cells [64, 66]. Recent evidence also suggests that Htt inclusion body formation deactivates apoptosis and results in slow necrotic cell death [67]. This contrasts with quality control partitioning of other aggregation-prone disease-associated proteins, such as SOD1, which accumulate in juxtanuclear compartments (JUNQ: JUxtaNuclear Quality control) for presentation to molecular chaperones for re-folding and/or the ubiquitin proteasome system for degradation [64, 66, 68, 69]. Therefore, findings pertaining to the HSR in the context of HD may uniquely apply to this ND due to the propensity of polyQ-expanded Htt to misfold directly into terminal aggregates and become stored in IPODs (as opposed to the JUNQ).

#### The HSR in striatal neurons

The expression and cellular accumulation of pathogenic polyQ-expanded Htt is not sufficient to induce an HSR in cell and animal models of HD. The expression of Htt^91Q^ does not induce an HSR (as assessed by an Hsp70 promoter driven EGFP expression construct) [70]. Notably, the expression of Htt^111Q^ in striatal cells was not sufficient to up-regulate Hsp expression nor activate HSF1 [32]. Likewise, primary cultures of rat cortical and striatal neurons expressing Htt^111Q^ showed low levels of Hsp70 mRNA transcripts and protein compared to cerebellar granule cells, which have high levels of Hsp70 and are resistant to degeneration [71]. Studies using transgenic mouse models of HD have demonstrated that there is a reduction in Hsp70 (and other molecular chaperones) in the brain as the disease progresses (Table 1) [72]. Taken together, these results show that cells expressing polyQ-expanded Htt do not sense or respond to this aggregation-prone protein by inducing an HSR.

**Table 1:** List of Hsps and whether their expression is up-regulated (↑), down-regulated (↓), or not changed (No ∆) across rodent models of HD compared to transgenic WT or non-transgenic mouse controls

Hsps	Transgenic disease models	Tissue or cell type	Reference
HSF1	STHdh(Q111) knock-in mice	IB: 80% ↓striatal and cerebellar tissue homogenates	[32, 74]
αB-c (HSPB5)	R6/2	IB: No Δ whole brain homogenates	[72]
	Htt-N171-82Q	IB: No Δ spinal cord homogenates	[79]
Hsp25 (HSPB1)	R6/2	IB: No Δ whole brain homogenates	[72]
	Htt-N171-82Q	IB: No Δ spinal cord homogenates	[79]
Hsp40	R6/2	IB: 60% ↓Hdj1 whole brain homogenates IB: 25% ↓Hdj2 whole brain homogenates	[72]
Hsp60	R6/2	LC-MS: 4-fold ↓ in protein abundance in the cortex LC-MS: 4-fold ↑ in protein abundance in the striatum	[80]
Hsp70	STHdh(Q111) knock-in mice	IB: 80% ↓striatal and cerebellar tissue homogenates	[32]
	R6/2	IB: ↓ whole brain homogenates IB: No Δ Hsc70 whole brain homogenates	[72]
Hsp90	R6/2	IB: No Δ Hsp90 whole brain homogenates IB: No Δ Hsp84 whole brain homogenates	[72]
Hsp105	–	–	–

These results are from immunoblot (IB), or liquid chromatography coupled with quantitative mass spectrometry (LC-MS) of affected CNS regions

The expression of polyQ-expanded Htt sensitizes neurons to heat stress [32, 73]. Heat shock (42 °C for 6 h) of primary murine striatal neurons expressing pathogenic Htt^111Q^ resulted in a 6-fold increase in caspase activity compared to heat shocked cells expressing non-pathogenic Htt^7Q^ [32]. Furthermore, cells expressing Htt^111Q^ had a reduced capacity to express Hsp70, Hsp25 (the mouse orthologue of Hsp27) and Hsp90 after heat shock compared to those expressing Htt^7Q^ [32]. These findings suggest that expression of polyQ-expanded Htt attenuates the capacity of striatal neurons to up-regulate the expression of Hsps after heat shock, which is normally a very strong activator of the HSR.

**Fig. 3:**
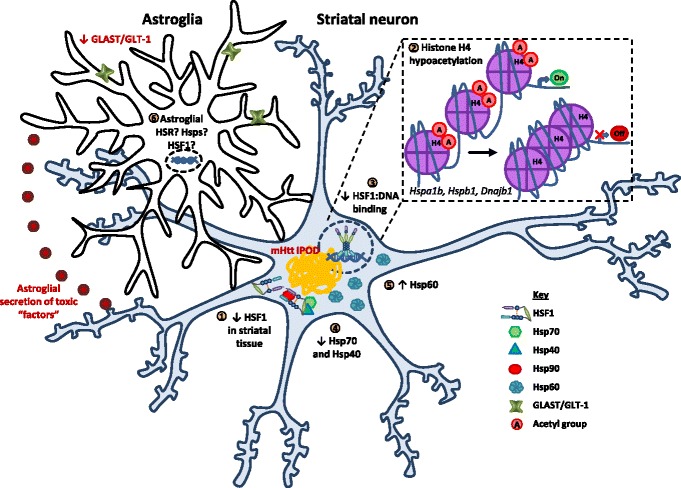
Proposed summary of changes in the HSR and its components in polyQ-expanded Htt over-expression models of HD. Huntington’s disease onset and progression into late stage is dependent on the molecular pathologies developed in striatal neurons (e.g. formation of polyQ-expanded Htt aggregates or IPODs) and astroglia (e.g. decline in GLAST/GLT-1 expression and the secretion of unidentified toxic “factors”). The susceptibility of striatal neurons to degeneration from HD-associated stresses could be the result of a polyQ-expanded Htt-mediated attenuation of the HSR. Over-expression of polyQ-expanded Htt in CNS tissues results in a (1) reduction in HSF1 levels. (2) Histone H4 acetylation has been shown to be a strong promoter of HSF1 binding to DNA of target genes. However, hypoacetylation of histone H4 at HSF1 targets (e.g. *Hspa1b, Hspb1,* and *Dnajb1*) with disease progression can explain (3) the decrease in HSF1 binding to DNA observed in polyQ-expanded Htt expressing striatal neurons. (4) HD disease progression is also associated with a decline in Hsp70 and Hsp40 family members and (5) a striatal-specific increase in Hsp60. (6) There have been few investigations regarding HSF1 activation and DNA-binding in astroglia. Therefore, the capacity of polyQ-expanded Htt over-expressing astroglia to activate HSF1 and induce an HSR is currently unknown

Based on these findings, it is pertinent to question whether the observed inability of striatal neurons to induce an HSR in the context of HD is the result of insufficient levels of HSF1, a failure to activate HSF1, a lack of HSF1 binding to DNA, or a combination of these deficiencies (Fig. 3). Quantification of total HSF1 protein by immunoblot demonstrated that primary striatal neurons expressing Htt^111Q^, and the striata and cerebella of HD mouse models, have lower total HSF1 levels compared to controls [32]. New evidence implicates CK2α’ kinase and Fbxw7 Fbox protein (an E3 ubiquitin ligase) in the enhanced degradation of HSF1 in cells expressing polyQ-expanded Htt [74]. The proposed model suggests that polyQ-expanded Htt up-regulates CK2α’ kinase expression and increases the phosphorylation of HSF1 at S303 and S307. This in turn recruits Fbxw7, which ubiquitinylates phospho-HSF1 targeting it for degradation by the ubiquitin proteasome system [74]. Enhanced degradation of HSF1 by the proteasome has also been implicated in a mouse model and human post-mortem tissues of α-synucleinopathies, whereby elevated levels of the ubiquitin ligase, NEDD4, ubiquitinates HSF1 for degradation [25]. Whilst expression of Htt^111Q^ is not sufficient to induce HSF1 activation in cerebellar granule cells, heat shock does result in activated (hyperphosphorylated) HSF1 trimers accumulating in the cell nucleus [32, 71]. Similarly, HSF1 dissociation from Hsp90, hyperphosphorylation, and nuclear translocation are not impaired in HD mice upon treatment with the Hsp90 inhibitor NVP-HSP990, which is able to penetrate into the brain [71]. Therefore, there is a reduction in total HSF1 levels in the striatum; however, HSF1 can become hyperphosphorylated and translocate into the nucleus in HD models.

With regard to the DNA binding capacity of HSF1 in HD models, evidence suggests that the expression of polyQ-expanded Htt attenuates HSF1 binding to DNA in striatal cells [73]. Genome-wide chromatin immunoprecipitation (ChIP) experiments demonstrated that in primary striatal neurons expressing Htt^111Q^, HSF1 is only capable of binding 39% of its target genes after heat shock (1159 bound genes) compared to cells expressing Htt^7Q^ (2943 bound genes) [73]. Microarray data show that the reduced HSF1 binding to DNA in heat shocked cells expressing Htt^111Q^ corresponds with a decline in the transcription of several Hsps (*Dndjb5*, *Dnajb12* and *Hspb6*). Furthermore, mRNA levels of Hsp70, Hsp40 and Hsp25 were reduced in the cerebral cortices of HD mouse models compared to controls following treatment with NVP-HSP990 [75]. Therefore, since HSF1 activation appears to be unimpaired, low levels of HSF1 and its compromised ability to bind DNA could account for the observed inability of cells expressing polyQ-expanded Htt to elicit an HSR. This dysregulation of chaperone gene expression likely plays a key role in the pathology of HD.

Altered chromatin architecture in HD may explain the reduced capacity of HSF1 to bind to target genes in striatal neurons under stress [75–78]. Quantitative PCR coupled with ChIP analysis after NVP-HSP990 (Hsp90 inhibitor) treatment demonstrated significant hypoacetylation of histone H4 at *Hspa1b, Hspb1, Dnajb1* genes in Htt^111Q^ expressing primary striatal neurons compared to Htt^7Q^ [75]. Hypoacetylation of these Hsp genes may change the chromatin landscape sufficiently to interfere with HSF1 binding to DNA (Fig. 3). Stress-inducible binding of HSF1 to DNA is associated with histone acetylation, H3K4 trimethylation, RNA polymerase II and other co-activators [77]. Furthermore, tetra-acetyl histone H4 has previously been suggested to be a strong modulator of HSF1 binding to DNA [77]. Therefore, it follows that histone H4 hypoacetylation in HD models may reduce chromatin accessibility of HSF1 at heat shock genes, consequently impairing the HSR.

At the tissue level, there are several studies that have investigated Hsp expression, and therefore HSR activation, in affected and non-affected tissues in mouse models of HD (Table 1). Immunoblot analysis of whole brain or spinal cord homogenates from HD mice have shown that overall there is no change in the total protein levels of the sHsps Hsp25 or αB-crystallin (αB-c, HSPB5) or Hsp90 family members compared to mice expressing non-pathogenic Htt [72, 79]. Whole tissue homogenates from HD mice showed a reduction in HSF1, Hsp70, and two Hsp40 family members (Hdj1 and Hdj2; Fig. 3) [32, 72]. Conversely, striatal homogenates from HD mice were found to have a 4-fold increase in Hsp60 levels compared to control mice [80]. A lack of immunohistochemistry in these studies makes it impossible to determine (i) where changes occur at the cellular level and (ii), whether small cell populations or specific cell-types induce an HSR, which is undetectable in bulk cell analyses. Employing immunohistochemistry in parallel to this work, or more powerful omics-based single-cell analyses, such as the proteomics approach undertaken by Sharma et al. [81], has the potential to resolve cell-type and brain-region specific differences in the presence or absence of disease. In this way, cell-type specific differences in the ability to induce a pro-survival HSR will be identified.

#### The role of glial cells in HD

There is evidence that the onset and progression of HD is both cell-autonomous and non-cell autonomous. One study showed that a transgenic mouse with pan-neuronal expression of Htt^103Q^ developed pathologies associated with end-stage disease, including astrogliosis, motor deficits and neurodegeneration [82, 83]. However, when Htt^103Q^ expression was restricted to either striatal neurons or cortical pyramidal neurons, pathologies associated with end-stage disease did not develop despite these cells having nuclear polyQ-expanded Htt aggregates and alterations in NMDA receptor function [82, 83]. These studies showed for the first time that HD pathogenesis and propagation depends on interactions between neuronal subtypes. Neuron-astroglia interactions have also been implicated in the non-cell autonomous progression of HD. A study that investigated transgenic mice that expressed Htt^98Q^ in both neurons and astroglia observed that these mice displayed more severe neurological symptoms and earlier death compared to mice in which Htt^98Q^ expression was restricted to either neurons or astroglia alone [84]. Therefore, it seems likely that both neuron-neuron and neuron-astroglia interactions are responsible for the onset and progression of HD.

The combination of molecular pathologies that develop in neurons and astroglia work in tandem to progress HD (Fig. 3). However, the mechanisms that underlie astroglial-mediated toxicity in HD are only partially understood. Astroglia expressing polyQ-expanded Htt secrete neurotoxic factors and decrease the expression of the glutamate transporters, GLAST and GLT-1 [85–87]. Indeed, polyQ-expanded Htt-expressing astroglia have been shown to increase the vulnerability of striatal and cortical neurons in co-culture to excitotoxic stresses [88]. Conversely, in co-culture experiments, astrocytes expressing non-pathogenic Htt^23Q^ protected 78% of the Htt^130Q^-expressing cortical neurons from glutamate toxicity [88]. Furthermore, conditioned medium derived from WT astroglial cultures protects Htt^111Q^-expressing striatal neuronal progenitor cells from neurotoxic insults (e.g. H_2_O_2_, glutamate, 3-nitropropionic acid) [89].

With respect to neurotoxic factors secreted by astroglia, an elegant study by Liddelow et al. [86], demonstrated that pro-inflammatory microglia can activate a neurotoxic phenotype in astroglia (defined in the work as A1 astroglia). They showed that A1 astrocyte formation is a pathological response of the CNS in mice treated with systemic injections of lipopolysaccharide and acute CNS injury, and in patients with NDs [86]. The proportion of neurotoxic (A1) astroglia in the caudate nucleus of HD patients was significantly greater than in controls (60% in HD tissue compared to 25% in the control) [86]. Furthermore, qPCR of A1 astroglia-associated transcripts showed a 60-fold increase in the caudate nucleus of HD patients compared to controls [86]. Thus, cell-cell interactions and dysfunctions of striatal or cortical pyramidal neurons and astroglia are likely to work synergistically to progress HD into late-stages. Therefore, it is important to maintain astrocytes in the striatum in a healthy and neurotrophic condition to minimize neurodegeneration in the striatum. The focus of research investigating pro-survival mechanisms and the proteostasis network in HD has thus far been predominantly focused on striatal neurons. However, considering accumulating evidence that implicates astroglia in the pathogenesis of HD, it is relevant to also investigate these pathways in astroglia.

#### The HSR in striatal astrocytes

Studies investigating HSR-inducing compounds have provided valuable insights into the capacity of different cell populations to induce an HSR in the absence of the expression of polyQ-expanded Htt. Primary striatal astrocytes derived from WT mice showed a stronger induction of the HSR compared to striatal projection neurons after treatment with the Hsp90 inhibitor, NVP-HSP990: treated striatal astroglia showed an 18-fold increase in Hsp70 and 4-fold increase in Hsp25 mRNA levels [90]. In contrast, NVP-HSP990 treated striatal projection neurons only showed an 8-fold increase in Hsp70, 3-fold increase in Hsp40, and no change in Hsp25 mRNA levels [90]. In the vehicle treated mice, there were no significant differences compared to non-treated mice in the synthesis of any of the Hsp mRNAs investigated (Hsp70, Hsp40, Hsp90 or HSF1), with the exception of Hsp25, which showed a 7-fold increase in astroglia compared to striatal projection neurons [90]. Together these findings suggest that, in the absence of polyQ-expanded Htt expression, striatal astroglia are “pre-loaded” with greater levels of Hsp25 mRNA and can induce a stronger HSR compared to striatal projection neurons.

The size and frequency of formation of polyQ-expanded Htt inclusions varies across different cell-types in the CNS. Striatal and frontal cortex glial fibrillary acidic protein (GFAP)-positive astroglia demonstrated a significant reduction in the size and the proportion of cells with Htt^210Q^ inclusions compared to neurons in these CNS regions in a mouse model of HD [91]. This difference was attributed to a possible increase in proteostasis network components (including heat shock proteins) in reactive astroglia. However, there is insufficient evidence from models of HD, with regards to the Hsp content of striatal astroglia, to draw relationships between inclusion size and number and proteostasis capacity of striatal astroglia (Fig. 3).

Determining mechanisms that maintain astroglia in a healthy and neurotrophic state should be a priority in future HD research, given that polyQ-expanded Htt expression decreases the levels of glutamate transporters and induces a neurotoxic phenotype of astroglia. Indeed, a recent study demonstrated that the over-expression of the sHsp, αB-c, in astroglia ameliorates the pathologies associated with HD in transgenic mice that express full-length Htt^97Q^ [92]. Over-expression of αB-c in astroglia significantly reduced the number of large (> 1 μm) Htt^97Q^ inclusions in the striatum and cortex, and resulted in a 14% increase in the number of NeuN-positive neurons in the striatum [92]. This provides an elegant example of how increasing Hsps in astroglia can ameliorate HD neuropathologies in a non-cell autonomous manner and provides support for increased investigation into the HSR in astroglia in the context of HD.

### Summary of the HSR in HD

It has been demonstrated using a range of cell and animal models of HD that striatal neurons do not sense or respond to polyQ-expanded Htt expression by up-regulating Hsps. Moreover, striatal neurons expressing polyQ-expanded Htt have an attenuated capacity to induce an HSR after heat shock. This could be due to decreased levels of HSF1 in striatal tissue observed in HD models. In addition, the altered chromatin landscape caused by histone H4 hypoacetylation at Hsp genes observed in a HD mouse model, may also lead to decreased binding of HSF1 to DNA, and down-regulation of Hsp70 and Hsp40. In the absence of polyQ-expanded Htt expression, striatal astroglia, compared to striatal projection neurons, have greater levels of Hsp25 mRNA and can induce a stronger HSR. However, the effect of polyQ-expanded Htt on HSR induction in astroglia remains to be elucidated.

Previous research has demonstrated that a variety of Hsps can inhibit polyQ-expanded Htt protein aggregation. However, the affected neurons in HD appear not to sense the initial stages of pathogenic protein misfolding as a cellular stress and therefore do not activate an HSR. As the neurons and surrounding astroglia in the CNS appear to be incapable of activating an HSR in the context of HD, therapeutics that can induce Hsp expression in early stages of disease may prove beneficial.

### Amyotrophic lateral sclerosis

Amyotrophic lateral sclerosis is characterized by a loss of motor neurons in the primary motor cortex, corticospinal tracts, brainstem and spinal cord. Only 5–10% of ALS cases are familial (fALS) and some of these arise from mutations in one of 13 (or more) genes leading to the expression of aberrant aggregation-prone proteins. Mutations in *SOD1* (copper/zinc ion-binding superoxide dismutase), *FUS* (fused in sarcoma), *TDP-43* (TAR DNA binding protein), *CCNF* (cyclin F) [93], *OPTN* (optineurin), *ANG* (angiogenin, ribonuclease, RNase A family, 5), *UBQLN2* (ubiquitin-like ubiquilin2), and others, as well as repeat expansions in *C9ORF72* (chromosome 9 open reading frame 72), are all associated with fALS (recently reviewed by [94, 95]). The remaining 90–95% of ALS cases are idiopathic and sporadic. In ALS, motor neurons are selectively susceptible to degeneration as a consequence of mutant ALS-associated protein expression, despite the ubiquitous presence of these same mutant forms in neuronal and non-neuronal cells [96]. Motor neurons have functional and morphological characteristics that may make them particularly vulnerable to toxic protein misfolding and aggregation, neuroinflammation, excitotoxic insults, oxidative damage and subsequent degeneration in ALS [96]. For example, motor neurons have a high metabolic load, high energy demands, long axons, and rely on rapid signaling of neurotransmitters to other neurons and muscle tissue by axonal transport. Malfunction of each of these characteristics of motor neurons have been associated with ALS pathology (for reviews see [97–99]). In addition, recent findings have shown that multiple housekeeping and stress-response pathways involved in maintaining proteostasis (e.g. the ubiquitin proteasome system and endoplasmic reticulum unfolded protein response) are dysregulated in ALS-affected motor neurons, which would further exacerbate their degeneration [96, 100–105]. However, it remains to be established whether affected cell-types in the CNS can induce an HSR as a result of pathogenic protein aggregation associated with ALS as a protective response to this chronic proteotoxic stress.

#### The HSR in motor neurons

Motor neurons may have a relatively high threshold for HSR induction compared to surrounding non-neuronal cells. Batulan et al. [53] investigated the endogenous expression of HSF1 in motor neurons and their capacity to activate the HSR. In these studies, immunohistochemistry of motor neurons in spinal cord cultures demonstrated the presence of HSF1, but not Hsp70, in these cells. The capacity of motor neurons to induce an HSR was assessed following heat shock (42 °C for 1 h) by monitoring the stress-inducible expression of an Hsp70 promoter driven EGFP construct. The lack of EGFP fluorescence in motor neurons after heat shock suggested that HSF1 was not activated and thus did not bind to the HSE used in this reporter construct [53]. Other studies have also shown that spinal cord motor neurons in situ fail to transcribe and express Hsp70 following heat shock [58, 106]. However, it is not clear from this work whether HSF1 was (i) not activated and therefore not capable of binding DNA and/or (ii) not present at sufficient levels in these cells to induce an HSR.

**Fig. 4:**
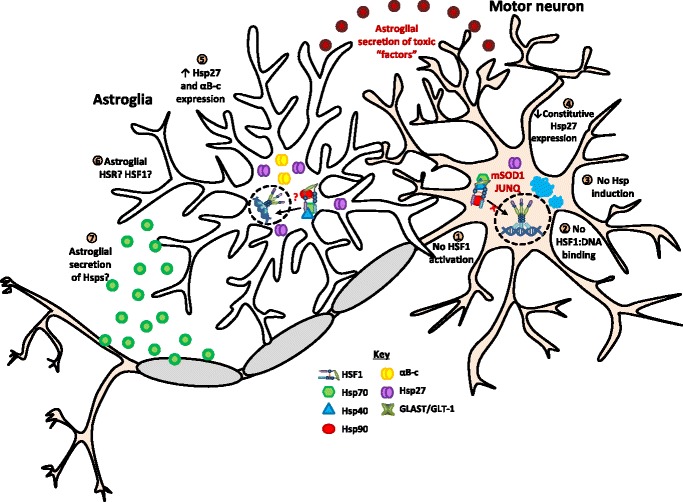
Proposed mechanism of the HSR and its components in motor neurons and astroglia of mSOD1 over-expressing models of ALS. Motor neuron disease initiation and progression is dependent on the molecular pathologies developed in motor neurons (e.g. formation of mSOD1 aggregates or JUNQ) and astroglia (e.g. secretion of unidentified toxic “factors”). The susceptibility of motor neurons to degeneration is likely due to an inability of motor neurons and astroglia to induce a cytoprotective HSR in response to increasing quantities of misfolded proteins (e.g. mSOD1). (1 and 2) HSF1 in motor neurons has a relatively high threshold for activation and the over-expression of mSOD1 and subsequent molecular pathologies do not activate HSF1 nor induce HSF1 binding to DNA. (3) There is no detectable basal expression or up-regulation of Hsps in mSOD1 over-expressing motor neurons, with the exception of (4) Hsp27, which gradually declines with disease progression. (5) Astroglia in mSOD1 over-expressing mice have increased amounts of Hsp27 and αB-c with disease progression; however, the levels of other Hsps are not changed. (6) There have been few investigations regarding HSF1 activation and DNA-binding in astroglia in the context of ALS. Therefore, the capacity of mSOD1 over-expressing astroglia to activate HSF1 and induce an HSR remains unknown. (7) There is increasing evidence that extracellular vesicles containing Hsps are secreted by astroglia, and these vesicles are endocytosed by motor neurons and facilitate transfer of Hsps

To determine which of these possibilities was responsible for these observations, WT HSF1 (HSF1^WT^) and a constitutively active mutant of HSF1 (HSF1^+^) were over-expressed in primary murine motor neurons [53]. Simply increasing the level of HSF1^WT^ in cells was not sufficient to enhance their capacity to express Hsp70 [53]. Conversely, over-expression of HSF1^+^ resulted in an up-regulation of Hsp70, Hsp40, and Hsp25 levels in 96.1 ± 3.4%, 100 ± 0%, and 14.6 ± 7.3% in motor neurons, respectively [53, 107]. Furthermore, expression of HSF1^+^ in motor neurons expressing pathogenic SOD1^G93A^ significantly reduced the formation of inclusions and conferred cytoprotection, compared to HSF1^WT^ [107]. Together, these findings suggest that the attenuated capacity of motor neurons to induce an HSR in the context of heat shock is due to their inability to activate HSF1, not insufficient levels of HSF1 in these cells (Fig. 4).

The over expression and aggregation of mutant SOD1 (mSOD1) in mouse models may not be sufficient to induce an HSR in motor neurons. Primary murine motor neurons expressing mSOD1 demonstrate no change in expression levels of Hsp70, Hsp105, Hsp90, Hsp60, or Hsp40 compared to those expressing SOD1^WT^ and non-transgenic control motor neurons (Table 2). Furthermore, a reduction in Hsp105 levels was observed in spinal cord tissue homogenates of SOD1^G93A^ mice [108]. Hsp105 is a chaperone expressed in neurons and glia- in the CNS and can inhibit the aggregation of mSOD1 in cell-based models [108]. Therefore, the decline in Hsp105 expression, combined with the inability of rodent motor neurons to up-regulate Hsp70, Hsp90, Hsp60, or Hsp40, suggests that the HSR is either unable to be activated or is impaired in these models of ALS (Fig. 4).

**Table 2:** List of Hsps and whether their expression is up-regulated (↑), down-regulated (↓), or not changed (No ∆) in rodent models of motor neuron disease at the late-stage of disease compared to age-matched transgenic WT or non-transgenic mouse controls

Hsps	Transgenic disease models	Tissue or cell type	Reference
HSF1	TDP-43^WT×Q331K^	IB: ↓ spinal cord tissue homogenates	[117]
	SOD1^G93A^	IB: ↓ spinal cord tissue homogenates	[174]
αB-c (HSPB5)	SOD1^L126Z^	IHC: ↑ spinal cord astroglia IHC: ↓ spinal cord oligodendrocytes	[115]
	SOD1^G93A^	IHC: ↑ spinal cord astroglia	[116, 175]
	SOD1^G37R^	IHC: ↑ spinal cord astroglia	[116]
Hsp22 (HSPB8)	SOD1^G93A^	IHC: ↑ spinal cord motor neurons	[176, 177]
Hsp25 (HSPB1)	SOD1^G93A^	IHC: ↑ spinal cord astroglia IHC: ↓ spinal cord oligodendrocytes	[53, 113, 114, 116]
	SOD1^G37R^	IHC: ↑ astroglia in the inferior colliculus, cerebellar white matter, brain stem, spinal cord	[79]
	SOD1^L126Z^	IHC: ↓ spinal cord motor neurons	[115]
	SOD1^G93A^_,_ SOD1^G85R^, SOD1^G37R^, SOD1^H46R/H48Q^	IHC: ↑ spinal cord astroglia IHC: ↑ spinal cord undefined neurons	[178]
Hsp40	SOD1^G93A^	IHC: No ∆ spinal cord	[79]
	SOD1^G93A^_,_ SOD1^G85R^, SOD1^G37R^, SOD1^H46R/H48Q^	IB: No ∆ spinal cord tissue homogenates	[178]
	TDP-43^WT×Q331K^	IB: No ∆ spinal cord tissue homogenates	[117]
Hsp60	SOD1^G93A^	IHC: No ∆ spinal cord	[79]
	SOD1^G93A^_,_ SOD1^G85R^, SOD1^G37R^, SOD1^H46R/H48Q^	IB: No ∆ spinal cord tissue homogenates	[178]
Hsp70	SOD1^G93A^_,_ SOD1^G85R^, SOD1^G37R^, SOD1^H46R/H48Q^	IB: No ∆ spinal cord tissue homogenates	[53, 79, 115, 178]
	SOD1^G93A^	IHC: ↑ “sick-appearing” spinal cord motor neurons	[179]
	SOD1^G85R^	IB: ↑ spinal cord tissue homogenates	[145]
Hsp90	SOD1^G93A^	IHC: No ∆ spinal cord	[79]
	SOD1^G93A^_,_ SOD1^G85R^, SOD1^G37R^, SOD1^H46R/H48Q^	IB: No ∆ spinal cord tissue homogenates	[178]
Hsp105	SOD1^G93A^	IB: ↓ in spinal cord tissue homogenates	[108]

These results are from immunoblot (IB) or immunohistochemical (IHC) staining and microscopy of different CNS regions

In the absence of disease, the endogenous and constitutive expression of Hsp27 in motor neurons of the spinal cord plays an important role in the housekeeping of proteostasis. Hsp27 is a well characterized molecular chaperone that also has potent anti-apoptotic functions [109]. Hsp27 can inhibit the release of mitochondrial cytochrome c and associate with Daxx, thereby inhibiting a motor neuron-specific apoptosis pathway mediated by Fas-Ask1-p38 [109–112]. However, in transgenic mouse models Hsp25 levels become dysregulated and decline in motor neurons during ALS progression (Table 2). Studies have shown that pre-symptomatic mSOD1 mice have Hsp25 levels comparable to age-matched controls [113, 114]. However, immediately prior to the onset of disease symptoms, Hsp25 levels decline in spinal cord motor neurons as demonstrated by immunohistochemistry of spinal cord sections [113, 114]. In contrast, by the late stages of the disease, both Hsp25 and αB-c are up-regulated in spinal cord astroglia, a finding consistent in numerous studies [79, 113–116]. Future research could address whether this decline in Hsp25 in mSOD1 expressing motor neurons correlates with an increase in Hsp25 sequestered in insoluble protein deposits. This could be achieved by immunoblot analysis of soluble and insoluble protein fractions and immunohistochemistry. The decrease in Hsp25 in motor neurons during the later stages of ALS in these transgenic mouse models may increase their susceptibility to neurodegeneration as a consequence of toxic protein accumulation and other ALS-related stresses (Fig. 4).

With regards to ALS in humans, immunohistochemistry of human motor neurons in cervical spinal cord sections obtained at autopsy showed no change in Hsp70 or Hsp27 levels compared to age-matched controls [53]. The combined evidence suggests that the toxicity of mSOD1 in spinal cord motor neurons is not sufficient to elicit the stress-inducible expression of Hsps in rodents or humans. In contrast, Hsp70 (but not Hsp27) immunoreactivity was occasionally observed to be higher in neighboring glial cells in fALS or sporadic ALS patients [53]. This suggests that astroglia in humans and rodents are capable of up-regulating certain Hsps in response to stresses associated with ALS. This further supports the hypothesis that motor neurons intrinsically have a high threshold for induction of the HSR and suggests that misfolded mSOD1 can go undetected by this inducible arm of the proteostasis network in these cells.

There is a lack of diversity in the models that have been used in work investigating the HSR in association with ALS. Data regarding neuronal and glial Hsp expression in ALS is derived primarily from mSOD1 rodent models, with the exception of one study that used the TDP-43^WT×Q331K^ transgenic mouse model of ALS [117]. Therefore, it remains to be determined whether these findings also apply to other fALS-associated mutations (e.g. *FUS*). This is of particular relevance if each aggregation-prone protein engages a specific set of Hsps, as has been previously proposed [118]. Therefore, additional research is required in other rodent models of ALS to advance our understanding of the HSR in this disease.

#### The role of glial cells in ALS

There is strong evidence that ALS can be characterized as a non-cell autonomous disease [99, 119]. As such, ALS initiation and progression depends on both the molecular pathologies developed within motor neurons, and the subsequent reactivity of surrounding non-neuronal cell populations such as astroglia and microglia. For example, transgenic mice expressing SOD1^G37R^ specifically in motor neurons in the ventral horn of spinal cords remained healthy for up to 1.5 years of age compared to mice that ubiquitously express this mSOD1 isoform, which die at 4 months of age [120, 121]. Moreover, knock out of SOD1^G37R^ expression in motor and dorsal root ganglion neuron progenitors of transgenic mice results in an 18 day delay to disease onset and 31 day delay to early disease progression compared to controls [122]. Together, these findings demonstrate that mSOD1 expression in motor neurons plays a role in early disease initiation and mSOD1 expression in other cells is required for disease progression. In support of this, selective deletion of SOD1^G37R^ expression from GFAP-positive spinal cord astroglia or cluster of differentiation molecule 11B (CD11b)-positive microglia did not slow disease onset or early disease progression, but significantly delayed late disease progression resulting in an overall extension of survival by 60 and 99 days, respectively [122, 123]. These two studies demonstrate that mSOD1 expression in astroglia and microglia plays a significant role in late disease progression and overall survival. This work and the work of many others emphasizes the importance of astroglia and microglia in the pathogenic cascade associated with ALS (for in-depth reviews examining the non-cell-autonomous nature of ALS see [99, 119, 124]).

The non-cell-autonomous progression of ALS by glial cells has also recently been demonstrated in a mutant TDP-43 model of ALS [125]. In a TDP-43^Q331K^ expressing transgenic mouse model of ALS, cre recombinase excision of the TDP-43^Q331K^ transgene from motor neurons completely rescued motor neuron death [125]. However, despite the increase in motor neuron survival, there was no significant difference in the number of degenerating axons and neuromuscular junctions or glial-mediated neuroinflammation in end-stage disease mice when the TDP-43^Q331K^ transgene was excised in motor neurons [125]. These findings are supported by research that showed that astroglial-specific expression of TDP-43^M337V^ in rats results in the death of spinal cord motor neurons and denervation of skeletal muscles [126]. One proposed mechanism for the neurotoxicity of astroglia in mutant TDP-43 expressing models is that neurotrophic genes are suppressed and neurotoxic genes are up-regulated (e.g. *LCN2* and *CHI3L1*) [127]. Combined, these studies suggest that the expression of mutant TDP-43 in astroglia is sufficient to cause non-cell-autonomous death of motor neurons. Furthermore, the non-cell-autonomous progression of ALS is not confined to mSOD1-expressing mouse models, but may represent a generic mode of ALS progression.

The ubiquitous expression of mutant ALS-associated protein in the CNS may not alone facilitate the transition of astroglia from a neurotrophic to neurotoxic phenotype in ALS. Co-culture with A1 astrocytes induces the rapid death of a range of neurons, including spinal cord α-motor neurons [86]. Interestingly, there was a significant increase in the proportion of A1 astroglia in the motor cortex of patients with ALS (40% of ALS astroglia were A1 compared to 15% in controls) [86]. Likewise, there was a 60-fold increase in A1-related transcripts in the motor cortex of ALS patients compared to controls [86]. The mechanism by which these A1 astrocytes induce toxicity was proposed to be through the secretion of a ‘toxic factor’ [86]. Other studies have also suggested that mSOD1-expressing astrocytes release a soluble ‘toxic factor’, which significantly reduces the viability of motor neurons in co-culture [128–131]. The identity of this neurotoxic factor is currently unknown and additional research is required to determine its mode of action. In any case, it is important to consider cytoprotective mechanisms that maintain the neurotrophic functions of glia in ALS, such as the HSR leading to Hsp expression.

#### The HSR in astroglia

It is generally regarded that astroglia are capable of activating an HSR in response to stress, including whole animal hyperthermia [51, 57–59]. In the context of ALS, astroglia have higher levels of the sHsps, αB-c and Hsp25, compared to WT controls at the end-stage of disease, but not Hsp90, Hsp70, Hsp60 or Hsp40 (Table 2, Fig. 4). Interestingly, these findings suggest that the over-expression of unstable and misfolded mSOD1 species in the CNS fails to activate the HSR. Moreover, the expression of sHsps and other Hsp families may not be under the same transcriptional and translational controls.

In astroglia, there is a scarcity of published work investigating HSF1-activation and HSR induction at the molecular level using biochemical techniques (Fig. 4). However, the discord between sHsps being up-regulated and other Hsps not being affected in astroglia in the context of ALS suggests that there are additional layers of regulation of the HSR in these cells that are either HSF1-mediated or post-translational. In recent work, Zheng et al. [28] hypothesized that the phosphorylation of HSF1 at serine and threonine residues serves to fine-tune HSF1 transcription at promoter regions, rather than acting solely as an on/off switch. Thus, HSF1 phosphorylation could serve to regulate the kinetics and magnitude of the HSR in a cell-type dependent manner. Additional unidentified mechanisms of HSF1 regulation, including those that are cell-type specific, could explain the complete absence of HSR induction in motor neurons compared to astroglia in mSOD1-expressing transgenic mice. In fact, the HSF1-mediated HSR in the different cell-types that comprise the CNS is likely to be much more complex than our current models of HSR induction and attenuation (Fig. 2), which are based primarily on findings from S*accharomyces cerevisiae*, *Drosophila melanogaster,* cell-lines or studies using recombinant human HSF1 in solution [38, 132–134]. Future research should elucidate mechanisms of HSR induction in astroglia, particularly astroglia that are affected in the spinal cord in ALS.

Knowledge of the precise mechanism by which HSF1 mediates the induction of specific Hsps in astroglia is important as it may uncover new therapeutic targets for the rescue of motor neurons from degeneration associated with ALS progression. This knowledge could be harnessed to up-regulate specific sets of Hsps that have been shown to interact with aggregating proteins associated with ALS [118]. For example, Hsp70 and HspB8 interact with TDP-43 and Hsc70, Hsp70, DNAJB1, DNAJB2a/b, Hsp27, αB-c, and HspB8 interact with SOD1 to prevent protein aggregation and decrease cytotoxicity [118]. It has been proposed that other Hsps are also likely to prevent the aggregation of TDP-43 and SOD1 since there are numerous ‘non-canonical’ members of the Hsp families that have not been tested for anti-aggregation or anti-apoptotic activities in these assays [118]. Up-regulation of cytoprotective Hsps in astroglia could maintain them in a healthy neurotrophic state to support motor neuronal viability and prevent conversion of astroglia to a neurotoxic (A1) phenotype. However, further research is needed to investigate mechanisms of HSF1-mediated HSR induction across different CNS cell-types in the presence and absence of disease-associated protein aggregation.

One mechanism by which astroglia may provide cytoprotection to motor neurons is through the exchange of extracellular vesicles containing Hsps [135]. Extracellular vesicles derived from chick spinal cord primary astroglial cultures following heat shock contain Hsp70 and Hsc70 [136]. In another study investigating glial-neuronal interactions, T98G glioma cells were shown to secrete Hsp70 into the culture medium and LA-N-5 neuroblastoma cells took up this Hsp70 [137]. The Hsp70 uptake increased the stress tolerance of the LA-N-5 cells to heat shock and staurosporine-induced apoptosis [137]. It would be interesting to extend on this work to investigate whether extracellular vesicles from more physiologically relevant cell-models (e.g. primary murine astroglia and motor neurons) also facilitate the trafficking of Hsps. The mechanisms of astroglial exocytosis and neuronal endocytosis used to traffic Hsps are also yet to be elucidated. This non-cell-autonomous mechanism(s) by which astroglia provide products of the HSR to neurons could be exploited to increase Hsp levels in motor neurons. This strategy could enhance the stress tolerance of motor neurons and decrease degeneration in the spinal cord in ALS (Fig. 4).

### Summary of the HSR in ALS

Spinal cord motor neurons from primary cell or animal models of ALS are unable to activate HSF1 and hence lack a stress-induced up-regulation of Hsps. The levels of Hsp27 in motor neurons decline with disease progression. Motor neurons have an inherently high threshold for the activation of the HSR and the expression and accumulation of mSOD1 in the cell is not sufficient to activate the HSR. In contrast, spinal cord astroglia have elevated levels of αB-c and Hsp25 (rodent) or Hsp70 (human) at the end-stage of disease. However, the precise mechanisms by which these Hsps are up-regulated are unknown. Overall, there is a distinct lack of research into the HSR in spinal cord astroglia and its potential role in ALS.

Due to the non-cell-autonomous nature of ALS, future research should focus on maintaining affected spinal cord astroglia in a neurotrophic state to support motor neuron viability. Furthermore, investigation of Hsp70 (and other Hsp) transfer between astroglia and motor neurons could represent an exciting new mechanism for the development of therapeutics that target the proteostasis network in ALS.

## Studying the therapeutic effects of increasing HSR components

The plaques and inclusions that are characteristic of ALS, HD and other NDs all share common morphological and biochemical features and this points to the highly related nature of these diseases [138, 139]. In addition, plaques and inclusion bodies are co-localized with various components of the proteostasis network, which may represent an irreversible sequestration and subsequent loss of function of these vital housekeeping components [140]. The sequestration of these chaperones, in conjunction with the possibility that toxic misfolded proteins are not sufficient to induce an HSR in the CNS, are likely to be important molecular mechanisms that lead to neurodegeneration in these diseases. However, there appears to be mechanistic differences in the way that these pathological proteins inhibit or evade detection by the HSR. In the case of Htt, there is evidence to suggest that the misfolded proteins themselves may directly impair mechanisms of the proteostasis network by changing the chromatin landscape (Fig. 3). The absence of a stress-induced up-regulation of Hsps in early disease allows the formation of toxic protein species, which precede a cascade of cellular dysfunctions in NDs. Therefore, in the absence of an HSR in affected neurons and surrounding glia in the CNS, boosting the HSR pharmacologically represents a promising therapeutic intervention for the treatment of these diseases at an early stage.

A significant amount of work has investigated the effects of over-expressing individual Hsps or activating an HSF1-mediated HSR in rodent models of NDs (for comprehensive reviews see [9, 36, 140–144]). Determining which of the HSR components are the most efficacious in preventing protein aggregation and subsequent neurotoxicity is an important step in elucidating targets for the development of therapeutics that ameliorate NDs. Over-expression of individual chaperones in mSOD1 mouse models of ALS has resulted in modest effects with regards to a reduction in the amount of insoluble protein and increased motor neuron survival (Table 3) [145–150]. However, this does not correlate with an increase in overall survival of the double transgenic animals (Table 3) [145–150]. Conversely, up-regulation of the HSR by treatment with withaferin A, celastrol or arimoclomol results in an increase in the number of surviving motor neurons and the lifespan of mSOD1 expressing mice [151–154]. This same trend was observed in mouse models of HD, whereby over-expression of HSJ1a and Hsp70 has no effect on overall survival but over-expression of an active mutant of HSF1 extended survival by 15 days (Table 4) [155]. The exceptions to this are DNAJB1, DNAJB6 and QBP-Hsc70 binding motif which, when over-expressed, were capable of reducing insoluble Htt and extending survival by 17, 21 and 32 days, respectively [156–159]. These findings illustrate how specific sets of Hsps may be more efficacious against aggregating proteins associated with HD. Furthermore, molecules that activate HSF1 and up-regulate the HSR (and therefore increase expression of a broad range of stress-related proteins) appear to be more capable of reducing protein aggregate load, preventing neurodegeneration and increasing lifespan of mouse models of NDs compared to up-regulation of individual chaperones.

**Table 3:** The effect of the over-expression of Hsps and up-regulation of the HSR on the molecular pathologies developed in rodent models of MND

Transgenic model/Therapeutic compound	MND model	Increase in Hsp in Tg mouse	Extended lifespan	% ↑/↓ in surviving motor neurons	% ↑/↓ in levels of inclusions	References
Hsp27 Tg	SOD1^G93A^	40-fold ↑spinal cord 25-fold ↑ cortex, cerebellum, hippocampus Expressed in MN + GFAP^+ve^ astroglia	No ∆ (prolonged 4.2 days)	–	No ∆	[146]
	SOD1^G93A^	–	No ∆ (died 6 days sooner)	24% ↑	No ∆	[147]
HSJ1a Tg	SOD1^G93A^	7-fold ↑	No ∆	61% ↑	No ∆	[148]
Hsp70 Tg	SOD1^G93A^	10-fold ↑	No ∆ (prolonged 1.4 days)	–	–	[145]
	SOD1^G85R^	10-fold ↑ spinal cord	No ∆	–	–	
	SOD1^G37R^	10-fold ↑	No ∆	–	–	
Hsp70 administered exogenously	SOD1^G93A^	rhHsp70 injected 3× weekly (20μg)- detected in muscle not CNS	9 days	12.5% ↑	–	[149]
HSF1 Tg	SOD1^H46R/H48Q^	3-fold ↑	No ∆	–	34% ↓	[151]
SIRT1 Tg	SOD1^G93A^	3-fold ↑	15 days	–	40% ↓	[150]
Withaferin A	SOD1^G93A^	2.6-fold ↑ Hsp25 2.2-fold ↑ Hsp70 Phosphorylated HSF1	8 days	30% ↑	39% ↓	[152]
	SOD1^G37R^	–	18 days	–	–	
Celastrol	SOD1^G93A^	–	16 days	30% ↑	–	[153]
Arimoclomol	SOD1^G93A^	3-fold ↑ Hsp70 2.5-fold ↑ Hsp90 Phosphorylated HSF1	28 days	74% ↑	–	[154]
NXD30001	SOD1^G93A^	No ↑ in Hsps in the CNS ↑ Hsp70 in skeletal muscle	–	–	–	[170]

Double transgenic (Tg) mice were bred for the over-expression of an Hsp and a SOD1 mutant associated with ALS. Alternatively, mice that over-express mSOD1 were treated with a therapeutic compound for the activation of the HSR. The fold increase in Hsp levels (and, if reported, the tissue-type in which this occurs), number of extended days of life, percent increase (↑) or decrease (↓) in spinal cord motor neurons, and percent ↑ or ↓ in the levels of inclusions is reported for each study

**Table 4:** The effect of the over-expression of Hsps and up-regulation of the HSR on the molecular pathologies developed in rodent models of HD

Transgenic model/Therapeutic compound	HD model	Increase in Hsp in Tg mouse	Extended lifespan	% ↑/↓ in surviving neurons	% ↑/↓ in levels of inclusions	References
αB-c Tg (astroglia only)	BACHD	–	–	12.5% ↑	50% ↓	[92]
Hsp27 Tg	R6/2	12-fold ↑	–	–	No ∆	[180]
Hsp70 Tg	R6/2	Rat Hsp70	–	–	No ∆	[72]
		5–15-fold ↑ human Hsp70	No ∆	No ∆	No ∆	[181]
rAAV-QBP1-Hsc70 binding motif	R6/2	Injected into the striatum	32 days	–	90.8% ↓	[158]
rAAV-DNAJB1	R6/2	Injected into the striatum	17 days	–	39.2% ↓	[159]
DNAJB6 Tg	R6/2	Brain-specific up-regulation (nestin promoter)	21 days	–	33% ↓	[157]
HSJa Tg	R6/2	Brain specific up-regulation	No ∆	No ∆	35% ↓	[182]
Hsp104	N171-82Q HD	“Strongly” expressed in the brain, heart kidneys, testis	–	–	No ∆	[183]
HSF1^Active^ Tg	R6/2	Expressed in skeletal muscle, heart and testes	15 days	No ∆	79% ↓	[155]
NVP-HSP990 treatment	R6/2	2.7-fold ↑ Hsp70 3.8-fold ↑ Hsp25 1.6-fold ↑ Hsp40	No ∆	–	20% ↓	[75]
HSF1 KO	R6/2	–	105 day decrease in lifespan	–	15% ↑	[184]
HSF2 KO	R6/2	–	91 day decrease in lifespan	–	20% ↑	[185]

Double transgenic (Tg) mice were bred for the over-expression of an Hsp and polyQ-expanded Htt associated with HD. Alternatively, HSF1 and HSF2 genes were knocked-out (KO) of HD mouse models. Lentiviral vectors for the expression of QBP1-Hsc70 binding motif and DNAJB1 were injected directly into the striatum of R6/2 mice. In one case, mice that over-express polyQ-expanded Htt were treated with NVP-HSP990, a therapeutic compound for the activation of the HSR. The fold increase in Hsp levels (and, if reported, the tissue-type in which this occurs), number of extended days of life, percent increase (↑) or decrease (↓) in spinal cord motor neurons, and percent ↑ or ↓ in the levels of inclusions is reported for each study

Investigations into the therapeutic benefit of pharmacological activation of the HSR in the context of NDs are currently in progress. There are two classes of therapeutics under investigation, each target different aspects of the HSR pathway. One class of therapeutics activate HSF1 and/or up-regulate downstream products of the HSR. These include celastrol, arimoclomol, withaferin A, acetyl, L-carnitine and pyrrolidine dithiocarbamate [160–164]. Thus far, arimoclomol is the most promising HSR-mediating therapeutic. Administration of arimoclomol to mouse models of ALS (10 mg/kg/day), spinal and bulbar muscular atrophy (120 mg/kg/day) and inclusion body myositis (120 mg/kg/day) ameliorated neuropathologies associated with each disease, and arimoclomol successfully passed phase I human clinical trials in 2008 [154, 165–167]. However, there is currently limited information regarding that status of phase II/III trials of arimoclomol in ALS patients.

Another class of therapeutics targets the HSF1 inhibitory complex composed of Hsp90, co-chaperone p23 and immunophilin FK506-binding protein 5. Since, Hsp90 activities are ATP-dependent, this complex can be targeted by small molecules that compete with ATP for binding to Hsp90. Radicicol, NVP-HSP990, geldanamycin and geldanamycin-derived 17-allylaminogeldanamycin are Hsp90 inhibitors that act in this way and have been investigated for the treatment of NDs [72, 75, 168, 169]. In HD mouse models, NVP-HSP990-induced induction of the HSR in CNS tissues but this effect declined with increasing age of mice and despite a 20% decline in inclusion load, no extension of life was observed [75]. Furthermore, in ALS mouse models, NXD30001 failed to induce the HSR in the CNS, despite the brain permeability of this molecule [170]. In vivo assessments investigating the efficacy of these compounds in the induction of the HSR has led to the conclusion that Hsp90 inhibitors are cytotoxic and not promising candidates to pursue for clinical trials.

A novel molecule identified in a yeast-based high-throughput screen, HSF1A, activates HSF1 by interacting with components of the inhibitory TRiC/CCT complex [171]. Treatment of cells with HSF1A results in HSF1 nuclear accumulation, trimerization, and enhanced binding to DNA [171]. Treatment of PC12 cells expressing polyQ-expanded Htt with HSF1A resulted in the reduction of inclusion bodies formed and protected cells against toxicity [171]. Furthermore, recent evidence shows that treatment of Neuro-2a cells transfected to express TDP-43-ΔNLS-K145Q with HSF1A results in a ~ 60% reduction in the number of aggregates formed compared to the vehicle control [172]. This indicates that the activation of endogenous HSF1 by HSF1A is sufficient to suppress mutant TDP-43 expression in this cell-based model [172]; however, it remains to be established whether HSF1A provides protection in vivo*,* in transgenic mouse models of NDs.

The therapeutic strategy of activating HSF1 in the CNS in the context of NDs is a promising one. Over 3 decades of research has provided strong in vitro evidence that activating HSF1 can reduce protein inclusion formation and ameliorate other molecular pathologies associated with a range of NDs [155, 172, 173]. However, progress towards the development of therapeutic compounds, that activate the HSR in affected tissues and cell-types in NDs has been slow. This is the likely due to the complexity of HSR induction in vivo, for example, in CNS tissues, Hsps may be provided to neurons in a non-cell autonomous manner that cannot be assessed in initial drug screens that are performed in vitro. Drug screens need to move beyond simple cell-based models and begin to incorporate more advanced tissue culture approaches to consider the responses from the multiple cell types present in tissues.

## Conclusions

This review has highlighted the complexity of the HSF1-mediated HSR and demonstrated that the generic model for its induction and attenuation does not take into account additional layers of regulation that are stress- and cell-type specific. This review highlights that regulation in the induction of the HSR differs not only between cell types (e.g. neurons and astroglia) but also between neuronal sub-types in different regions of the brain (e.g. striatal neurons and motor neurons). It is these regulatory aspects of the HSR that are of particular interest with regards to the cells that comprise the CNS, where protein inclusion formation associated with NDs occurs. We propose that the appearance of protein inclusions in discrete neuronal populations in NDs may result from low (or no) basal levels of Hsps and/or a high threshold of HSR induction in these cells. Indeed, two recent findings in an α-synucleinopathy and HD suggest a possible generic underlying pathology in NDs, whereby HSF1 is targeted for degradation by the proteasome through elevated expression or activity of ubiquitinases [25, 74]. Furthermore, evidence from a range of models of NDs indicates that the species formed during protein misfolding and aggregation do not cause sufficient cellular stress to induce an HSR in affected neurons and astroglia. This suggests that cell-autonomous and non-cell-autonomous mechanisms that maintain protein homeostasis are insufficient to prevent protein aggregation associated with NDs in the CNS and highlights that the HSR is a promising pathway to target in the development of novel therapeutics for these diseases.

Future research should investigate basal and stress-inducible HSR proteins in neurons and astroglia, preferably using quantitative, high throughput and single-cell analysis techniques, which circumvent contamination by other cell types. This is particularly important due to the growing body of evidence that implicates neuronal-astroglial interactions in the progression of ALS and HD into late- and end-stage disease (as is also the case for other NDs). In addition, it is important to investigate the mechanisms by which astroglia can be maintained in a neurotrophic (rather than neurotoxic) phenotype in NDs. In compiling this review, it highlighted to us that there is a scarcity of research that has investigated the HSR in astroglia in the context of ALS or HD (and indeed most NDs). Furthermore, the ability of astroglia to provide non-cell-autonomous support to neurons by trafficking Hsps and other neurotrophic factors in exosomes is currently understudied in physiologically relevant models. Future research in this field should investigate the HSR in NDs by adopting a more holistic approach and focus on defining the cell-autonomous and non-cell-autonomous HSR within different cells-types of the CNS.

## Abbreviations

ALS: Amyotrophic lateral sclerosis
ANG: Angiogenin
C9ORF72: Chromosome 9 open reading frame 72
CCNF: Cyclin F
CD11b: Cluster of differentiation molecule 11B
ChIP: Chromatin immunoprecipitation
CNS: Central nervous system
DBD: DNA binding domain
DNA: Deoxyribonucleic acid
fALS: Familial amyotrophic lateral sclerosis
FUS: Fused in sarcoma
GFAP: Glial fibrillary acidic protein
GLAST: Glutamate aspartate transporter
GLT-1: Glutamate transporter
HD: Huntington’s disease
HSE: Heat shock element
HSF1: Heat shock transcription factor 1
Hsps: Heat shock proteins
HSR: Heat shock response
Htt: Huntingtin
IPOD: Perivacuolar inclusion bodies
JUNQ: Juxtanuclear compartments
KO: Knock-out
LZD: Leucine zipper domain
mRNA: Messenger ribonucleic acid
mSOD1: Mutant superoxide dismutase 1
NDs: Neurodegenerative diseases
NMDA: N-methyl-D-aspartate
OTPN: Optineurin
PCR: Polymerase chain reaction
PolyQ: Polyglutamine
QBP: Polyglutamine binding peptide
qPCR: Quantitative polymerase chain reaction
RD: Regulatory domain
RNA: Ribonucleic acid
sHsps: Small heat shock proteins
SOD1: Superoxide dismutase 1
SUMO: Small ubiquitin like modifier
TAD: Transactivation domain
TDP-43: TAR DNA binding protein
Tg: Transgenic
TRiC/CCT: T-complex protein-1 ring complex/chaperonin containing TCP1
UBQLN2: Ubiquilin 2
WT: Wild-type
αB-c: AlphaB-crystallin
